# Genetic Variants in PGE2 Receptors Modulate the Risk of Nephrosclerosis and Clinical Outcomes in These Patients

**DOI:** 10.3390/jpm11080772

**Published:** 2021-08-06

**Authors:** Luz María González, Nicolás Roberto Robles, Sonia Mota-Zamorano, José Manuel Valdivielso, Juan López-Gómez, Guillermo Gervasini

**Affiliations:** 1 Department of Medical and Surgical Therapeutics, Division of Pharmacology, Medical School, University of Extremadura, 06006 Badajoz, Spain; luzmariagg@unex.es (L.M.G.); smotazamorano@gmail.com (S.M.-Z.); 2 Service of Nephrology, Badajoz University Hospital, 06080 Badajoz, Spain; nrrobles@unex.es; 3 Vascular and Renal Translational Research Group, UDETMA, ISCIII REDinREN, IRBLleida, 25198 Lleida, Spain; valdivielso@irblleida.cat; 4 Service of Clinical Analyses, Badajoz University Hospital, 06080 Badajoz, Spain; lopezhospi@yahoo.es

**Keywords:** nephrosclerosis, PGE2, EP receptors, cardiovascular risk

## Abstract

Prostaglandin E2 (PGE2) is a major actor mediating renal injury. We aimed to determine genetic variability in the genes coding for its receptors (*PTGER1-4*) and study associations with nephrosclerosis risk and clinical outcomes. We identified 96 tag-SNPs capturing global variability in *PTGER1-4* and screened 1209 nephrosclerosis patients and controls. The effect of these variants was evaluated by multivariate regression analyses. Two *PTGER3* SNPs, rs11209730 and rs10399704, remained significant in a backward elimination regression model with other non-genetic variables (OR = 1.45 (1.07–1.95), *p* = 0.016 and OR = 0.71 (0.51–0.99), *p* = 0.041, respectively). In the nephrosclerosis patients, a proximal region of *PTGER3* was tagged as relevant for eGFR (*p* values for identified SNPs ranged from 0.0003 to 0.038). Two consecutive *PTGER3* SNPs, rs2284362 and rs2284363, significantly decreased systolic (*p* = 0.005 and *p* = 0.0005), diastolic (*p* = 0.039 and *p* = 0.005), and pulse pressure values (*p* = 0.038 and 0.014). Patients were followed for a median of 47 months (7–54) to evaluate cardiovascular (CV) risk. Cox regression analysis showed that carriers of the *PTGER1*rs2241360 T variant had better CV event-free survival than wild-type individuals (*p* = 0.029). In addition, *PTGER3*rs7533733 GG carriers had lower event-free survival than AA/AG patients (*p* = 0.011). Our results indicate that genetic variability in PGE2 receptors, particularly EP3, may be clinically relevant for nephrosclerosis and its associated CV risk.

## 1. Introduction

Chronic kidney disease (CKD), whose prevalence has increased by an alarming 30% in the last 30 years [1], is now present in approximately 10% of the population, making this disease a global healthcare issue that is predicted to be the fifth cause of death worldwide by 2040 [2]. Among the pathological processes involved, nephrosclerosis usually refers to chronic renal insufficiency in a hypertensive and/or aging patient in the absence of other renal pathologies [3]. Although progression to end-stage renal disease (ESRD) is uncommon, the impact of the disease on the global cardiovascular (CV) risk is a major concern [4].

Chronic reduction of prostaglandins in the kidney, such as that caused by non-steroidal anti-inflammatory drugs (NSAIDs), may result in hypertension [5]. In addition, NSAIDs have been shown to increase the incidence of CV problems, indicating the involvement of prostaglandins (PG) in the pathogenesis of CV diseases [6]. This background highlights the role of inflammation in nephrosclerosis. Thus, there are inflammatory changes in the initial stages of CKD and the control of inflammatory response is key to delaying kidney damage [7,8]. One of the most important inflammatory pathways is the cycloxygenase (COX)-mediated synthesis of PG, of which PGE2 is the main renal metabolite and a major actor mediating renal injury [9,10]. PGE2 actions are conducted through the activation of four different G-protein-coupled receptors (EP1-4), which may cause vasodilation/constriction and influence renal blood flow and hemodynamics [11]. These receptors are also involved in a variety of damaging mechanisms, such as hyperfiltration, fibrosis, apoptosis, oxidative stress, or inflammation [12].

The susceptibility to CKD is significantly influenced by genetics [13,14]; therefore, a plausible hypothesis is that the presence of functional variants in the genes that code for PGE2 receptors (*PTGER1-4*), given the aforementioned role of this PG, may favor the onset of nephrosclerosis and/or affect clinical outcomes. Despite the existence of a few reports linking some of these single nucleotide polymorphisms (SNPs) to hypertension [15,16] and acute coronary syndrome [17], their effect on CKD patients remains untested. Our aim was to examine patients diagnosed with nephrosclerosis and control subjects to determine whether variability in the four genes coding for PGE2 receptors (defined by tag-SNPs) may be associated with the risk of this disorder and/or clinical outcomes in these patients.

## 2. Results

Clinical and demographic characteristics of the population are shown in Table 1. The study included 1209 subjects, 716 controls, and 493 patients with nephrosclerosis (stage 3 or higher). Statistical differences between control and CKD groups were observed for age, sex, weight, BMI, hypertension, diabetes, hyperlipidemia, blood pressure, cholesterol, occurrence of CV events, creatinine, albumin-to-creatinin ratio, albuminuria, and eGFR. In addition, differences were also observed among CKD groups regarding age, ethnicity, weight, BMI, cholesterol, serum creatinine, albumin-to-creatinin ratio, albuminuria, and estimated glomerular filtration rate (eGFR) (Table 1).

### 2.1. Genetic Associations with the Risk of Nephrosclerosis

The overall call rate and reproducibility percentages of the genotyping analysis were 97.8 and 99.71, respectively. One SNP in *PTGER2* and six in *PTGER3* were not in Hardy–Weinberg equilibrium and hence they were ruled out from subsequent analyses.

The results of the univariate analyses testing associations between the remaining 89 SNPs and the risk of nephrosclerosis in all models of inheritance are shown in Supplementary Materials Table S1. After controlling for confounding variables, ten SNPs, all in the *PTGER3* gene, were significantly associated with the risk of the disease (Table 2).

The predictive power of the identified SNPs was evaluated with ROC curves in models including or not relevant non-genetic covariates. In spite of a slight increase in the AUC value (80.2% vs. 78.6%) in favor of the combined genetics/classic model, the difference was not statistically significant (*p* = 0.379, Supplementary Figure S1).

Next, we included these 10 SNPs together in an adjusted multiple regression model with backward elimination controlling for the aforementioned covariates. Four SNPs remained in the final model, two of which, rs11209730 and rs10399704, still showed a statistically significant association with the risk of nephrosclerosis (OR = 1.45 (1.07–1.95), *p* = 0.016 and OR = 0.71 (0.51–0.99), *p* = 0.041, respectively, Table 3).

### 2.2. Impact of Polymorphisms on Renal Function and Blood Pressure Traits

**Figure 1:**
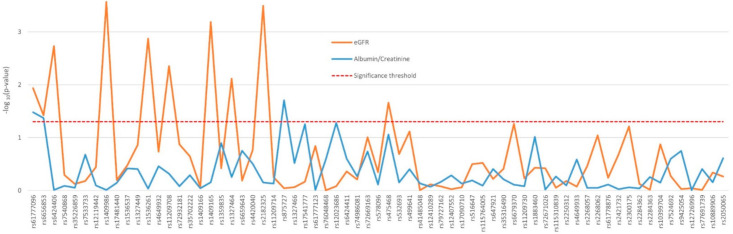
Effect of genetic variability in the *PTGER3* gene determined by tag-SNPs on the renal function of nephrosclerosis patients.

The influence of the 89 SNPs on the renal function of the 430 nephrosclerosis patients who were not in dialysis was analyzed adjusting by sex, age, ethnicity, hypertension, diabetes, and CKD stage. Ten variants in *PTGER3*, mainly located in the proximal region of the gene, showed a significant association with eGFR, with *p* values ranging from 0.0003 to 0.038 (Figure 1). Associations with the albumin-to-creatinine ratio were far less noticeable and also only observed in *PTGER3* (Figure 1). The two first variants in the gene, rs61777096 and rs6656853, were the only ones to significantly affect both eGFR and proteinuria. Mean eGFR and albuminuria values displayed by the patients with significant differences between genotypes are shown in Supplementary Table S2.

**Figure 2:**
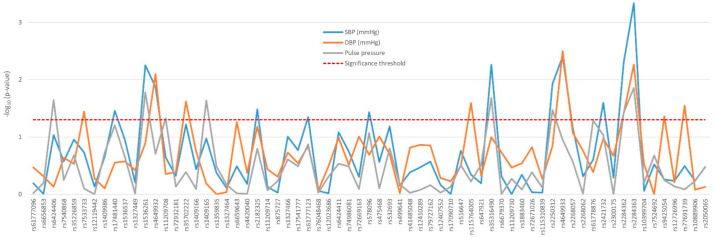
Effect of genetic variability in the *PTGER3* gene determined by tag-SNPs on blood pressure traits of nephrosclerosis patients.

Except for *PTGER4* rs16870224, all the variants found to affect blood pressure traits—namely, systolic (SBP) and diastolic blood pressure (DBP) and pulse pressure—were located in *PTGER3*, mostly in a distal area of the gene (Figure 2). Supplementary Table S3 shows the differences across genotypes observed in the nephrosclerosis patients. Most notably, two consecutive tag-SNPs, rs2284362 and rs2284363 were found to significantly decrease SBP (*p* = 0.005 and *p* = 0.0005, respectively), DBP (*p* = 0.039 and *p* = 0.005), and pulse pressure values (*p* = 0.038 and 0.014, Figure 2).

### 2.3. Association of PTGER Variants with the Incidence of Cardiovascular Events

Participants were followed for a median of 47 months (range 7–54), in which a total of 50 CV events were reported, nine in the control group (1.3%), and 41 (8.3%) in the nephrosclerosis patients (OR = 7.13 (3.4–14.8), *p* = 1.32 × 10^−9^). Among the CKD patients, those with CV events were significantly older and predominantly males. These and other characteristics of the patients experiencing or not CV events are summarized in Table 4.

**Figure 3:**
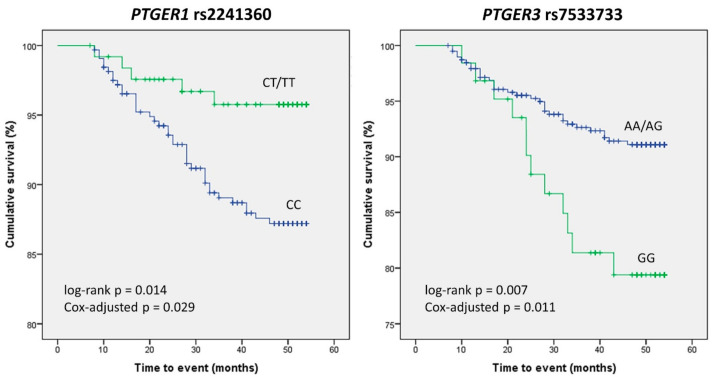
Kaplan–Meier curves depicting the occurrence of cardiovascular events in nephrosclerosis patients. Differences between genotypes of *PTGER1* rs2241360 and *PTGER3* rs7533733 are shown.

In order to narrow down the number of SNPs to be analyzed in the survival study, we carried out a previous analysis of the crude association of the 89 variants with the risk of experiencing a CV event under the selected genetic model. Five SNPs resulted in significant associations, with the rs7533733 GG genotype displaying the highest OR and the lowest *p* value (OR = 2.65 (1.28–5.46), *p* = 0.01, Table 5). Kaplan–Meier analysis of these five polymorphisms revealed that carriers of the *PTGER1* rs2241360 T variant allele had better CV event-free survival than homozygous wild-type carriers did (log-rank *p* = 0.014), whilst patients homozygous for the *PTGER3* rs7533733 G variant allele experienced more events than AA/AG carriers (log-rank *p* = 0.007, Figure 3). After adjusting these results in a Cox regression model accounting for other confounding variables (see Supplementary Table S4 for details), the differences between genotypes in both SNPs remained statistically significant (*p* = 0.029 and 0.011, respectively).

## 3. Discussion

The evolution of kidney damage in nephrosclerosis is usually slower than in diabetic nephropathy. However, the high prevalence of the disease and the great interindividual variability in its progression result in a significant proportion of cases experiencing a vicious cycle: kidney damage worsens CV risk, which, in turn, increases disease progression to ESRD [18,19]. There is therefore a need for identifying additional risk factors for nephrosclerosis that may allow early detection, the individualization of treatments and the reduction of the enormous economic burden caused by CKD and renal replacement therapy.

Our findings showed that nine tag-SNPs, each representing a haplotype block in the *PTGER3* gene locus, and rs2268062, also in *PTGER3*, which was included in the study for being reportedly linked to hypertension [15], were significantly associated with the risk of nephrosclerosis. Variability in the *PTGER3* gene locus has not been extensively studied, and consequently there is no data regarding the functional impact of polymorphisms. We do know, however, that the administration of EP3 antagonists in vivo increases COX-2-mediated PGE2 expression in the kidney, whilst the receptor activation decreases PGE2 levels [20]. This is relevant to the development of nephrosclerosis, as PGE2 contributes significantly to kidney disease, as it is involved in albuminuria, growth/fibrosis, and the activation of the renin–angiotensin–aldosterone system (RAAS) [21]. Therefore, it is tempting to speculate that the areas tagged by the identified variants might affect the function/expression of the receptor, which, in turn, would translate into altered levels of PGE2 in renal tissue, hence modifying the susceptibility to kidney damage. Another hypothesis is that the risk of CKD was modulated by the impact of genetic variability on the direct actions of EP3, e.g., vasoconstriction [22], water balance [23], regulation of renal blood flow [24], or maintenance of renal cell integrity [25]. Finally, changes in the susceptibility to nephrosclerosis could also be the result of changes in the susceptibility to the main risk factor, i.e., hypertension. As we mentioned above, the information about the clinical impact of these SNPs in *PTGER3* is very scarce. To our knowledge, there is only one report mentioning an association of rs2268062 with hypertension in the general population, although the observation could not be replicated in a validation sample [15]. In our sample, this SNP was not associated with hypertension, in fact, only rs2250312 in a recessive model appeared to be linked to this phenotype (data not shown). In any case, the fact that 95.7% of our nephrosclerosis patients had hypertension makes it much harder to identify genetic markers for this feature in this cohort than in a general population setting. On the other hand, the effect of the identified tag-SNPs was not profound enough to significantly improve a predictive model (calculated with ROC analysis) based on classic risk factors. The most probable reason is that the impact of nongenetic factors such as hypertension was as marked as to overshadow that of the genetic variants.

The study of the nephrosclerosis cohort identified several variants in the *PTGER3* gene associated with significant changes in renal parameters and blood pressure traits. In particular, the effect on eGFR was especially noticeable, with several SNPs tagging a proximal region of the gene (from genomic position 1:70854192 to 1:70905737) highly linked to altered filtration. Again, we can only speculate on the consequences of genetic variants in this area, but given that one of the main EP3 roles is to reduce hyperfiltration by constricting the afferent arteriole [11,26], it is more than likely that the presence of functional polymorphisms linked to these tag-SNPs may have a significant impact in eGFR values.

The study also tagged regions in *PTGER3* that were relevant for blood pressure. This is in line with previous in vitro data and animal studies. Thus, rodent models have shown that the administration of selective EP3 agonists results in an acute and significant rise in arterial pressure [27] and that the pressor actions of PGE2 are mediated by EP3 [28]. Furthermore, we found that only variants in *PTGER3* were relevant for blood pressure. Accordingly, it has been reported that the expression levels of EP3 in both renal resistance vessels and the aorta are much higher than those of other EP receptors, and that its vasopressor effects are far superior to EP2 and EP4 vasodilator properties [27]. Finally, another fact that highlights the relevance of some of the *PTGER3* SNPs identified in this study, and therefore that of the tagged regions, is that some of these variants were repeatedly pinpointed in both the risk analysis and the cohort study. Most notably, out of the 89 polymorphisms studied, rs11209708 was observed to increase the risk of nephrosclerosis as well as affecting eGFR and pulse pressure values. In any case, it should be mentioned that the overall effects of PGE2 that are mediated by EP receptors are particularly complex and depend on many factors aside from genetics—e.g., EP1–4 relative expression levels—additional hormonal signaling (angiotensin II, endothelin, etc.) or the individual clinical status [12].

CV mortality in nephrosclerosis is up to 20 times more frequent than in the general population [4], and even after renal replacement therapy, mortality rates are higher in patients with nephrosclerosis than in other CKD groups [18]. In this regard, two SNPs, located in the genes coding for EP1 and EP3, had a significant impact on the incidence of CV events in the nephrosclerosis cohort. There is ample evidence to support the hypothesis that changes in these genes can lead to an altered CV risk. For instance, the activation of these two receptors increases intracellular Ca^2+^ via phospholipase C, in opposition to EP2 or EP4, which have no effect on Ca^2+^. Voltage-dependent Ca^2+^ channels are widely distributed throughout the body and play a critical role in the maintenance of vascular tone. Indeed, substantial research has demonstrated the association between cardiovascular disease and the dysregulation of intracellular calcium [29,30,31]. Moreover, PGE2 is present in mouse atherosclerotic plaques, where it can potentiate platelet aggregation, an effect solely mediated by EP3 [32,33]. Indeed, mice lacking EP3 develop less severe thrombosis after administration of arachidonic acid [34] and atherothrombosis induced in vivo by mechanical rupture of the plaque is drastically decreased when platelets lack EP3 [32]. This background and the findings presented herein suggest that variability in the genes coding for EP3 and, to a lesser extent, EP1, may play a key role in the occurrence of CV events in nephrosclerosis patients.

This work has a number of limitations. First, a validation cohort was lacking; paradoxically, however, this was a consequence of one of the strengths of the study: the requirement of a specific nephrosclerosis diagnosis, which improved the homogeneity of the cohort by excluding patients with diabetic kidney disease. Another limitation is that the *PTGER3* gene was far more polymorphic than *PTGER1*, *2*, and *4* and therefore many more SNPs were needed to tag the entire gene locus. This could have resulted in more relevant results obtained. Finally, one limitation was inherent to the study design. We analyzed representative SNPs in a region of the genome with high linkage disequilibrium—i.e., their determination makes it possible to infer total genetic variability and identify phenotypic associations without genotyping the rest of SNPs in that area. However, there is a drawback, tag-SNPs are intronic variants and therefore we cannot speculate on their functional consequences as we could in the case, for instance, of a nonsynonymous polymorphism.

In a seminal review, Nasrallah et al. propose targeting PGE2 receptors as a potential new pharmacological mechanism to prevent kidney damage and dysfunction in CKD [12], although it is true that the actual benefits in vivo of this strategy are still a matter of debate [35]. To our knowledge, there are no previous reports on how the modulation of these PGE2 receptors could affect outcomes in renal patients. In this work, we have shown that genetic variability in the genes encoding for these receptors may indeed be relevant, not only for the susceptibility to nephrosclerosis, but also for several important phenotypic traits in these patients. Most importantly, given the high CV risk shown by individuals with the disease, we have identified two genetic variants in *PTGER1* and *PTGER3* that were solidly linked to the occurrence of CV events, including death. These findings strengthen the aforementioned hypothesis that PGE2 receptors may constitute valuable therapeutic targets in nephrosclerosis and point to certain areas in the genes loci that may be of interest in this regard. Notwithstanding, more data from independent cohorts, and especially in vitro studies that can characterize the functional consequences of SNPs in *PTGER*1-4, are warranted to confirm our findings.

## 4. Patients and Methods

### 4.1. Study Design

The study was designed as an observational, retrospective study on 493 patients diagnosed with nephrosclerosis and 716 controls. Patients’ samples were obtained from two sources: (i) the NEFRONA repository, which archives biological samples that were collected in a former multicenter study of cardiovascular morbidity and mortality in Spanish subjects with CKD stage 3 or higher (see explanation below), including patients in dialysis [36]; and (ii) the Nephrology Service of the Badajoz University Hospital, where patients with the same characteristics were recruited over a four-year period.

KDIGO (Kidney Disease—Improving Global Outcomes, kdigo.org, accessed on 4 August 2021) is a global nonprofit organization developing and implementing evidence-based clinical practice guidelines in kidney disease. According to its guidelines, CKD is defined as abnormalities of kidney structure or function, present for more than three months, with implications for health. CKD is divided into five stages based on levels of kidney function assessed by the glomerular filtration rate, which was estimated using the Modification of Diet in Renal Disease (MDRD) equation. These stages are Stage 1: Mild kidney damage, eGFR 90 mL/min/1.73 m^2^ or higher; Stage 2: Mild loss of kidney function, eGFR 60–89 mL/min/1.73 m^2^; Stage 3a and 3b: Mild to severe loss of kidney function, eGFR 30–59 mL/min/1.73 m^2^; Stage 4: Severe loss of kidney function, eG FR 15–29; and Stage 5: Kidney failure or close to failure, eGFR less than 15 mL/min/1.73 m^2^. Therefore, our patients (stage 3 and higher) had all an eGFR lower than 60 mL/min/1.73 m^2^.

Nephrosclerosis patients over 18 years of age were selected according to current diagnostic guidelines, i.e., those with biopsy alterations typical of vascular nephropathy or that met clinical criteria. These criteria were based on the absence of signs of other kidney diseases and the presence of data suggestive of the pathology (advanced age, long-standing hypertension, left ventricular hypertrophy, initially mild renal failure and proteinuria below 0.5–1 g/24 h). Patients with proteinuria higher than 1 g were biopsied to confirm the diagnosis. Proteinuria was defined as a value greater than 500 mg or albuminuria higher than 300 mg in 24 h urine. Diagnostic and prognostic stratification of patients were carried out using the KDIGO classification, the KDIGO table of risk of progression and the CONSORTIUM-CKD equation (Kidney Risk Failure; www.kidneyriskfailure.org, accessed on 4 August 2021). CV risk was defined as the likelihood of experiencing a fatal or non-fatal CV event in the four-year follow-up (54 months). CV events included acute myocardial infarction, acute coronary syndrome, coronary catheterization requiring angioplasty, coronary bypass, typical angina with positive stress tests, sudden death, cerebrovascular accident, peripheral arterial disease, aortic aneurysm, and lower limb ischemia.

Control subjects matched by sex and age with eGFR > 60 mL/min/1.73 m^2^ were recruited from: (i) Badajoz University Hospital; (ii) Primary Care centers throughout the country in the case of samples from the NEFRONA repository; and (iii) the DNA repository of the Instituto de Salud Carlos III (www.bancoadn.org, accessed on 4 August 2021). Exclusion criteria included previous history of any CV event, transplantation of any organ, carotid artery surgery, active infection, pregnancy or life expectancy below one year. All subjects gave written consent for their participation in the study, which had been approved by the Ethics Committee of the participating institutions, and that was carried out in accordance with the Declaration of Helsinki and its subsequent revisions.

### 4.2. SNP Selection and Genotype Analysis

We retrieved the coding sequence and adjacent 3′ and 5′ UTR regions of the *PTGER1* (ENSG00000160951; HGNC:HGNC9593), *PTGER2* (ENSG00000125384, HGNC:HGNC:9594), *PTGER3* (ENSG00000050628; HGNC:HGNC9595), and *PTGER4* (ENSG00000171522; HGNC:HGNC9596) genes, coding for EP1–4 receptors, and identified tag-SNPs (polymorphisms that represent genetic variability in a certain area) with Haploview 4.2 (Cambridge, MA, USA). In order to capture common variations, we chose a pair-wise tagging with a minimum r^2^ of 0.80 and a threshold for minor allele frequency of 10%. In addition to the tag-SNPs identified, we included two variants, rs17197 and rs2268062, with a reported impact on BP [15,16]. In this manner, 6 SNPs in *PTGER1*, 8 SNPs in *PTGER2*, 73 SNPs in *PTGER3*, and 9 SNPs in *PTGER4* were analyzed. The list of all evaluated SNPs is shown in Supplementary Table S1.

Whole blood samples (10 mL) were drawn from the participants recruited at the Badajoz University Hospital and stored at −80 °C until DNA purification, which was conducted with a standard phenol-chloroform extraction and ethanol precipitation. DNA samples were then stored at 4 °C in sterile plastic vials. In the case of participants recruited in the NEFRONA study, genetic material was obtained from biological samples stored at the REDinREN biobank [37] using QIAamp DNA Blood Kits (Hilden, Germany).

Genotyping was performed by allelic discrimination using TaqMan^®^ OpenArray Genotyping (Waltham, MA, USA) with a customized panel on a QuantStudio™ 12K Flex Real-Time PCR System (Life Technologies, Carlsbad, CA, USA) in the Centro Nacional de Genotipado-Instituto de Salud Carlos III (CeGen-ISCIII; Madrid, Spain, www.cegen.org, accessed on 4 August 2021). A trio of samples from the Coriell Institute biorepository, with known genotypes, were included in each chip as quality control.

### 4.3. Statistical Analyses

Differences between quantitative variables were assessed by the Student’s *t*/Mann–Whitney or ANOVA/Kruskal–Wallis tests, depending on the normality of the data and the number of groups compared. Categorical variables were compared with the Chi-square test. Logistic regression modeling was used to examine the influence of the SNPs on vascular CKD risk, adjusting for demographics and classic risk factors, namely age, sex, ethnicity, diabetes and hypertension, as formerly described [38]. After testing five inheritance models in the preliminary genetic analyses (codominant, dominant, recessive, overdominant, and log-additive), we decided to utilize the dominant model in the risk analysis, as we have done in former reports by our group [39,40], because the resulting genotype groups were the most balanced in terms of size and because it resulted in more significant associations. In order to examine the value of the studied SNPs in predicting susceptibility to CKD, receiving operating curves (ROC) were generated for models containing classic clinical and demographic risk factors adding or not the genetic information. The area under the curve (AUC) of these models were compared with the DeLong test. Genetic association analyses with clinical variables were performed with regression modelling adjusting by confounding variables.

The incidence of CV events and its association with the presence of SNPs was assessed by Kaplan–Meier curves, which were compared with the log-rank test. Cox regression modeling was carried out in order to evaluate the effect of additional covariates. Patients were followed up until the earliest of CV event, death, or end of study.

Statistical analyses were carried out with the *SNPassoc*, *pROC*, and *survival* packages in the R environment and the IBM SPSS statistical software (SPSS Inc., Chicago, IL, USA; version 22.0).

## 5. Conclusions

The determination of genetic variability of PGE2 receptors, and particularly that of EP3, may be useful to identify patients at risk of nephrosclerosis and/or the detection of patients with this disease that have a higher likelihood of experiencing CV events.

## Figures and Tables

**Table 1:** Clinical and demographic characteristics of the population of study. Median (range) or count (percentages) are shown.

	Control (*n* = 716)	CKD 3 (*n* = 307)	CKD 4–5 (*n* = 123)	CKD 5D (*n* = 63)	Total (*n* = 1209)	*p* Value (Control vs. CKD)	*p* Value (between CKD Groups)
Age (yrs)	60 (51–68)	67 (61–71)	64 (57–71)	61 (50–68)	62 (54–69)	6.2 × 10^–17^	1.05 × 10^–4^
Males (%)	359 (50.2)	211 (68.7)	80 (65.0)	43 (68.3)	693 (57.4)	7.87 × 10^–10^	0.758
Ethnicity							
Caucasian	709(99.0)	304 (99.0)	120 (97.6)	58 (92.1)	1191 (98.5)	0.065	0.003
Others	7 (1.0)	3 (1.0)	3 (2.4)	5 (7.9)	18 (1.5)		
Weight (kg)	75.2 (66–85.8)	80.3 (72–88.6)	78.6 (67.5–87.2)	75 (64.8–86)	77.3 (67.6–86.7)	1.1 × 10^–5^	0.030
BMI	30.4 (26.4–93)	30.3 (27.5–34.6)	28.8 (25.8–33.1)	27 (24.2–31.1)	30 (26.5–37.5)	1.6 × 10^–4^	7.0 × 10^–6^
Hypertension	405 (56.6)	293 (95.4)	121 (98.4)	60 (95.2)	879 (72.8)	1.95 × 10^–61^	0.333
DM	55 (7.7)	74 (24.1)	32 (26.0)	8 (12.7)	169 (14.0)	4.23 × 10^–14^	0.100
Hyperlipidemia	175 (35.9)	184 (67.2)	91 (74.0)	39 (61.9)	489 (51.6)	7.43 × 10^–24^	0.203
Smoking							
Non-smoker	328 (45.8)	124 (40.4)	53 (43.1)	26 (41.3)	531 (43.9)	0.255	0.929
Current-smoker	125 (17.5)	53 (17.3)	22 (17.9)	13 (20.6)	213 (17.6)		
Former-smoker	263 (36.7)	130 (42.3)	48 (39.0)	24 (38.1)	465 (38.5)		
Systolic blood pressure (mmHg)	136 (124–149)	145 (132–160)	146 (133.5–161)	139 (130–155)	139 (127–154)	3.08 × 10^–14^	0.162
Diastolic blood pressure (mmHg)	81 (74–88)	81 (75–89)	79 (73–88)	80 (70–90)	81 (74–88.5)	0.838	0.410
Pulse pressure (mmHg)	54 (46–64.5)	64 (51–77)	64 (55–76.5)	60 (50–70)	57 (48–70)	5.6 × 10^–20^	0.123
Total cholesterol (mg/dL)	200.7 (180–222.8)	185 (159.8–213.3)	175.5 (148.5–203)	160.5 (137.8–185.5)	192 (165–216)	1.9 × 10^–17^	1.9 × 10^–5^
Cardiovascular events	9 (1.3)	25 (8.1)	10 (8.1)	6 (9.5)	50 (4.1)	1.33 × 10^–9^	0.933
Creatinine (mg/dL)	0.8 (0.7–1)	1.5 (1.3–1.7)	2.9 (2.5–3.6)		1 (0.8–1.6)	1.06 × 10^–102^	3.09 × 10^–40^
Albumin/creatinine (mg/g)	6.4 (4–41.2)	37.1 (7.5–195.1)	212.1 (46–601)		33.4 (5.1–182.4)	1.27 × 10^–11^	3.6 × 10^–5^
eGFR (ml/min/1.73 m²)	88.8 (78.7–100)	45 (37.4–50.8)	21 (16.2–25.6)		71.2 (40.2–90.1)	2.31 × 10^–143^	4.39 × 10^–56^

BMI, body mass index; DM, diabetes mellitus; eGFR, estimated glomerular filtration rate.

**Table 2:** Association of *PTGER3* polymorphisms with the risk of nephrosclerosis. Odds ratios with 95% confidence intervals (OR) were adjusted by sex, age, ethnicity, diabetes and hypertension.

Polymorphism	Genotype	Control *n* (%)	CKD *n* (%)	OR	*p* Value
rs11209708	A/A	558 (80.5)	369 (77.4)	1.41 (1.01–1.97)	0.043
	A/G-G/G	135 (19.5)	108 (22.6)		
rs6424411	A/A	330 (48.0)	203 (42.7)	1.32 (1.01–1.74)	0.045
	A/G-G/G	358 (52.0)	272 (57.3)		
rs475468	G/G	254 (37.0)	207 (43.1)	0.72 (0.54–0.95)	0.018
	G/A-A/A	432 (63.0)	273 (56.9)		
rs6679370	A/A	201 (29.1)	166 (34.4)	0.71 (0.53–0.95)	0.022
	A/G-G/G	490 (70.9)	316 (65.6)		
rs11209730	A/A	475 (66.6)	306 (62.7)	1.38 (1.04–1.83)	0.024
	A/G-G/G	238 (33.4)	182 (37.3)		
rs2250312	C/C	311 (43.7)	191 (38.9)	1.41 (1.07–1.84)	0.013
	C/T-T/T	401 (56.3)	300 (61.1)		
rs2268062	T/T	209 (29.3)	171 (34.9)	0.69 (0.51–0.92)	0.011
	T/C-C/C	504 (70.7)	319 (65.1)		
rs61778876	A/A	646 (93.4)	460 (95.4)	0.54 (0.31–0.95)	0.03
	A/G	46 (6.6)	22 (4.6)		
rs2300175	C/C	207 (29.0)	170 (34.7)	0.69 (0.52–0.92)	0.012
	C/T-T/T	507 (71.0)	320 (65.3)		
rs10399704	G/G	163 (23.4)	139 (29.2)	0.72 (0.52–0.98)	0.035
	G/A-A/A	534 (76.6)	337 (70.8)		

**Table 3:** Multivariate logistic regression model for the risk of nephrosclerosis.

	B	SE	Wald	OR	CI	*p* Value
Sex	0.748	0.15	25.87	2.11	(1.58–2.82)	3.65 × 10^–7^
Diabetes	1.119	0.21	27.39	3.06	(2.01–4.65)	1.66 × 10^–7^
Hypertension	3.077	0.27	127.95	21.70	(12.73–36.98)	1.15 × 10^–29^
rs11209708 A/G-G/G	0.348	0.18	3.75	1.42	(1.00–2.01)	0.053
rs6424411 A/G-G/G	0.251	0.15	2.95	1.29	(0.97–1.71)	0.086
rs11209730 A/G-G/G	0.37	0.15	5.83	1.45	(1.07–1.95)	0.016
rs10399704 G/A-A/A	−0.345	0.17	4.16	0.71	(0.51–0.99)	0.041
Constant	−3.573	0.34	111.52	0.03		4.54 × 10^–26^

B, regression coefficient; SE, standard error; OR, odds ratio; CI, 95% confidence interval.

**Table 4:** Parameters of interest in nephrosclerosis patients experiencing or not cardiovascular events. Median (range) or count (percentages) are shown.

Variable	No CVE	CVE	*p* Value
Age (years)	66 (59–71)	67 (64–72.5)	0.014
Sex (male)	300 (66.4)	34 (82.9)	0.019
Ethnicity			
Caucasian	441 (97.6)	41 (100)	0.381
Others	11 (2.4)	0	
Weight	79.8 (69.7–88)	81.5 (70.9–91.5)	0.568
BMI (kg/m²)	29.8 (26.5–33.4)	29.7 (26.1–33)	0.923
Hypertension	435 (96.2)	39 (95.1)	0.480
DM	100 (22.1)	14 (34.1)	0.064
Hyperlipidemia	* 284 (67.8)	30 (73.2)	0.302
Smoking			
Non-smoker	190 (42.0)	13 (31.7)	0.224
Current-smoker	77 (17.0)	11 (26.8)	
Former-smoker	185 (49.9)	17 (41.5)	
Pulse pressure (mmHg)	62 (51–75.4)	67 (57.3–78.3)	0.096
Systolic lood pressure (mmHg)	144 (132–160)	145 (134–162)	0.642
Dyastolic blood pressure (mmHg)	81 (74–89)	77.5 (70.8–88.8)	0.245
Chronic kidney disease stage			
Stage 3	282 (62.4)	25 (61.0)	0.933
Stage 4–5	113 (25.0)	10 (24.4)	
Dialysis	57 12.6)	6 (14.6)	
Total cholesterol (mg/dL)	181 (154.7–209)	165 (139.5–212)	0.065
Creatinine (mg/dL)	1.7 (1.4–2.4)	1.7 (1.3–2.3)	0.689
Albumin/Cr (mg/g)	68.9 (9.9–294.3)	173.6 (25.5–470.6)	0.171
eGFR (mL/min/1.73 m²)	38.2 (26.6–47.7)	37.4 (25–47)	0.705
Glucose (mg/dL)	100.5 (90–114)	102 (96.5–123.5)	0.134
Calcium (mg/dL)	9.4 (9.1–9.7)	9.5 (9.1–9.7)	0.947
Sodium (mEq/L)	141 (139–142)	141 (139–142)	0.951
Potassium (mEq/L)	4.7 (4.4–5.1)	4.8 (4.4–5)	0.946

* Dyslipemia data was missing in 33 individuals.

**Table 5:** Crude risk analysis for the occurrence of cardiovascular events in nephrosclerosis patients.

Gene	Polymorphism	Model	Genotype	No CVE	CVE	OR	*p* Value
*PTGER1*	rs2241360	Dominant	CC	316 (70.9)	34 (85.0)	0.43 (0.18–1.05)	0.037
			CT/TT	130 (29.1)	6 (15.0)		
*PTGER3*	rs7533733	Recessive	AG/AA	390 (86.5)	29 (70.7)	2.65 (1.28–5.46)	0.010
			GG	61 (13.5)	12 (29.3)		
*PTGER3*	rs12119442	Dominant	G/G	304 (69.7)	21 (51.2)	2.19 (1.15–4.18)	0.014
			G/A-A/A	132 (30.3)	20 (48.8)		
*PTGER3*	rs74986081	Dominant	GG	333 (75.7)	34 (89.5)	0.37 (0.13–1.05)	0.035
			GA/AA	107 (24.3)	4 (10.5)		
*PTGER3*	rs2268057	Overdominant	CC/TT	254 (56.3)	16 (39.0)	2.02 (1.05–3.88)	0.025
			CT	197 (43.7)	25 (61.0)		

CVE, cardiovascular event; OR, odds ratio with 95% confidence interval.

## Data Availability

The data underlying this article will be shared on reasonable request to the corresponding author.

## Supplementary Materials

The following are available online at https://www.mdpi.com/article/10.3390/jpm11080772/s1, Figure S1. ROC curves analyzing the predictive power for a nephrosclerosis diagnosis of a combined model containing genetic and non-genetic variables (green curve) and a model including classic risk factors only (blue curve); Table S1. Univariate analysis of the association of *PTGER* polymorphisms with the risk of nephrosclerosis in each model of inheritance. Significant *p* values are in boldface type; Table S2. Differences in parameters of renal function shown by nephrosclerosis patients according to polymorphisms in the *PTGER3* gene; Table S3. Differences in blood pressure traits shown by nephrosclerosis patients according to polymorphisms in *PTGER* genes; Table S4. Cox regression analyses modelling the risk of cardiovascular events in nephrosclerosis patients according to relevant genotypes.
