# Supplementary material for: Genetic Variants in PGE2 Receptors Modulate the Risk of Nephrosclerosis and Clinical Outcomes in These Patients
Source: J Pers Med. 2021 Aug 6;11(8):772. doi: 10.3390/jpm11080772 (PMC8400263; doi:10.3390/jpm11080772)
Supplement: Supplementary file 1 [file jpm-11-00772-s001.zip › Supplementary tables.pdf]

SUPPLEMENTARY TABLES

Suppl Table S1. Univariate analysis of the association of *PTGER* polymorphisms with the risk of nephrosclerosis in each model of inheritance. Significant p-values are in boldface type.

		Model of inheritance				
	**SNP**	**codominant**	**dominant**	**recessive**	**overdominant**	**log-additive**
PTGER1	rs11668633	0.75884	0.77760	0.56719	0.54001	0.97301
	rs2241362	0.38724	0.61364	0.17423	0.90506	0.41556
	rs2241360	0.35318	0.18480	0.40117	0.27649	0.15263
	rs28364039	0.98331	0.93266	0.86195	0.95787	0.91098
	rs11671086	0.78023	0.48177	0.78103	0.61606	0.52344
	rs3760702	0.34895	0.93874	0.16229	0.37538	0.44732
PTGER2	rs111907219	0.62194	0.44091	0.71237	0.35015	0.58027
	rs1254585	0.56534	0.30258	0.98466	0.29732	0.39426
	rs17125344	0.99172	0.95941	0.91441	0.93908	0.98013
	rs708499	0.54298	0.58061	0.28866	0.84222	0.42006
	rs2229187	0.45431				
	rs17197	0.90490	0.87666	0.65897	0.96024	0.80745
	rs45461592	0.73140	0.76635	0.44089	0.87579	0.67454
PTGER3	rs61777096	0.69711	0.39873	0.72896	0.46631	0.40758
	rs6656853	0.22879	0.26813	0.10920	0.95147	0.11353
	rs6424406	0.69942	0.48404	0.57890	0.53235	0.44777
	rs7540868	0.33687	0.14452	0.49015	0.44038	0.17930
	rs35226859	0.14827	0.25387	0.06180	0.88256	0.07940
	rs7533733	0.80460	0.56478	0.62235	0.82791	0.50965

rs12119442	0.27631	0.11306	0.52493	0.15964	0.11223
rs1409986	0.39305	0.19057	0.56876	0.21833	0.17597
rs17481440	0.46690	0.21714	0.79410	0.23225	0.23142
rs1536537	0.13077	0.88165	0.06593	0.09026	0.31773
rs1327449	0.72680	0.78688	0.42534	0.95913	0.61560
rs1536261	0.33221	0.61459	0.13788	0.62693	0.27157
rs4649932	0.87691	0.99927	0.62422	0.80415	0.84596
rs11209708	0.06833	0.05055	0.38728	**0.02901**	0.09771
rs72932181	0.60720	0.32073	0.76690	0.34812	0.32085
rs35702222	0.94790	0.76571	0.83110	0.83286	0.74416
rs1409166	0.38018	0.33409	0.20797	0.90573	0.18711
rs1409165	0.25963	0.11621	0.39746	0.18475	0.10055
rs1359835	0.42374	0.27236	0.29296	0.85923	0.19013
rs1327464	0.27669	0.56706	0.21431	0.17828	0.88188
rs6659643	0.96984	0.95340	0.80472	0.99511	0.91687
rs4420040	0.32037	0.46678	0.13718	0.70245	0.19717
rs2182325	0.23612	0.13217	0.23142	0.41876	0.08954
rs11209714	0.56675	0.61848	0.29237	0.79483	0.37189
rs875727	0.66821	0.37167	0.92315	0.37485	0.39568
rs1327466	0.25617	0.17715	0.55090	0.10978	0.31932
rs17541777	0.25472	0.37025	0.25622	0.18695	0.65761
rs61777123	0.54331	0.89069	0.30042	0.65014	0.87944
rs76048468	0.60970	0.50621	0.51787	0.44425	0.57888
rs12023686	0.75478	0.48245	0.69256	0.53523	0.45771
rs6424411	0.07007	0.06182	0.46574	**0.02115**	0.27444
rs74986081	0.69970	0.64317	0.55120	0.52609	0.77881

rs72669163	0.69411	0.52405	0.67515	0.44803	0.62196
rs578096	0.67143	0.37613	0.90262	0.38490	0.41776
rs475468	0.06428	**0.02295**	0.21563	0.16649	**0.02331**
rs532693	0.25123	0.11875	0.86602	0.09665	0.17864
rs499641	0.58704	0.31336	0.58105	0.61299	0.33016
rs41485048	0.13497	0.19704	0.08793	0.32726	0.12428
rs12410289	0.51893	0.25246	0.76146	0.27864	0.26286
rs79727162	0.11491	0.12922	0.09334	0.24469	0.07575
rs12407552	0.20420	0.16023	0.50859	0.08265	0.34574
rs17090710	0.16870	0.14316	0.46418	0.06891	0.32270
rs516647	0.28204	0.37494	0.32104	0.13294	0.84872
rs115764005	0.21810	0.14728	0.26370	0.18311	0.12390
rs647921	0.90895	0.82414	0.76065	0.71858	0.93845
rs35316490	0.86223	0.96926	0.59227	0.89626	0.85546
rs6679370	0.12181	**0.04219**	0.36350	0.24804	0.06168
rs11209730	**0.02333**	**0.02177**	0.43136	**0.00676**	0.09749
rs1883460	0.34828	0.91857	0.18060	0.31168	0.55240
rs72671026	0.69156	0.39405	0.77983	0.42310	0.39273
rs115310839	0.78464	0.53011	0.85697	0.49656	0.58187
rs2250312	0.06308	**0.01990**	0.69822	**0.03909**	0.05357
rs4649933	0.91673	0.81295	0.79727	0.71230	0.92482
rs2268057	0.13837	0.05549	0.25932	0.23174	0.05086
rs2268062	**0.03888**	**0.01642**	0.11642	0.32514	**0.01290**
rs61778876	**0.03169**	**0.03169**			
rs2421732	0.47378	0.22621	0.63960	0.27419	0.22586
rs2300175	**0.04168**	**0.01782**	0.11766	0.34138	**0.01383**

	rs2284362	0.29046	0.16977	0.25978	0.42894	0.11758
	rs2284363	0.63556	0.34453	0.89586	0.35266	0.38221
	rs10399704	0.11879	**0.04310**	0.29370	0.39440	0.05880
	rs7524692	0.51014	0.25187	0.87162	0.27698	0.32421
	rs9425054	0.74068	0.88197	0.48235	0.70410	0.94807
	rs12726996	0.22548	0.17168	0.19937	0.26063	0.12404
	rs77691739	0.59552	0.73192	0.31902	0.85692	0.62521
	rs10889906	0.30440	0.40510	0.13581	0.94766	0.18879
	rs2050065	0.50005	0.37488	0.32742	0.61601	0.27366
PTGER4	rs13186505	0.92538	0.76625	0.73682	0.89993	0.70773
	rs10039983	0.82313	0.69794	0.73570	0.53470	0.93710
	rs2228058	0.24419	0.10065	0.51869	0.12583	0.09317
	rs4546432	0.50262	0.78514	0.24287	0.54388	0.41532
	rs11957406	0.71294	0.43401	0.99338	0.50069	0.62115
	rs56277923	0.67102	0.71742	0.48968	0.51409	0.93899
	rs13354346	0.78072	0.48208	0.79748	0.58897	0.52093
	rs28540420	0.40685	0.25019	0.74030	0.18082	0.41354
	rs16870224	0.44005	0.34890	0.27197	0.58682	0.24547

Supplementary Table S2. Differences in parameters of renal function shown by nephrosclerosis patients according to polymorphisms in the *PTGER3* gene.

Gene	SNP	Genotype	Mean (SE)	Mean diff.	p-value
			eGFR (ml/min/1.73 m2)		
PTGER3	rs61777096	G/G	36.95 (0.82)	2.11 (0.48 - 3.74)	0.012
		A/G-A/A	36.61 (1.24)		
	rs6656853	C/C	36.38 (1.07)	1.64 (0.09 - 3.18)	0.038
		T/C-T/T	37.20 (0.89)		
	rs6424406	T/T	37.38 (0.75)	-3.28 (-5.33 -1.23)	0.002
		C/T-C/C	34.55 (1.59)		
	rs1409986	G/G	37.21 (0.75)	-3.94 (-6.04 -1.84)	0.0006
		A/G-A/A	33.95 (1.64)		
	rs1536261	C/C	39.28 (1.06)	-2.52 (-4.04 -0.99)	0.001
		A/C-A/A	34.96 (0.88)		
	rs11209708	A/A	37.20 (0.78)	-2.63 (-4.43 -0.83)	0.004
		G/A-G/G	35.07 (1.39)		
	rs1409165	A/A	37.44 (0.83)	-2.86 (-4.49 -1.23)	0.0006
		G/A-G/G	35.06 (1.21)		
	rs1327464	G/G	35.78 (1.02)	2.07 (0.55 - 3.59)	0.008
		A/G-A/A	37.42 (0.93)		
	rs2182325	G/G	39.29 (0.99)	-2.81 (-4.33 -1.29)	0.0003
		A/G-A/A	35.20 (0.90)		
	rs475468	G/G	38.04 (1.05)	-1.81 (-3.34 -0.26)	0.022
		A/G-A/A	36.09 (0.89)		
			Albumin-to-creatinine ratio (mg/g)		
PTGER3	rs61777096	G/G	281.6 (45.26)	192.95 (16.57 - 369.30)	0.033
		A/G-A/A	460.6 (93.07)		
	rs6656853	C/C	250.6 (47.59)	170 (6.45 - 335.40)	0.043
		T/C-T/T	399.6 (64.45)		
	rs875727	T/T	296.04 (42.93)	263 (43.29 - 482.8)	0.019
		C/T-C/C	553.41 (133.01)		

SE, standard error; Mean diff., mean difference with 95% confidence interval

Supplementary Table S3. Differences in blood pressure traits shown by nephrosclerosis patients according to polymorphisms in *PTGER* genes.

Gene	SNP	Genotype	Mean (SE)	Mean diff.	p-value
		Sistolic blood pressure (SBP)			
PTGER3	rs17481440	C/C	145.4 (1.03)	5.54 (0.4 10.67)	0.035
		C/T-T/T	151.1 (2.09)		
	rs1536261	C/C	149.6 (1.49)	-5.43 (-9.26 -1.61)	0.006
		C/A-A/A	144.2 (1.23)		
	rs4649932	A/A	148.4 (1.29)	-4.72 (-8.44 -0.99)	0.013
		A/G-G/G	143.5 (1.39)		
	rs2182325	G/G	148.4 (1.39)	-4.05 (-7.75 -0.34)	0.033
		G/A-A/A	144.3 (1.27)		
	rs61777123	T/T	145.1 (1.06)	4.45 (0.11 - 8.79)	0.045
		T/G-G/G	149.5 (2.04)		
	rs578096	C/C	145 (1.08)	4.33 (0.27 - 8.38)	0.037
		C/T-T/T	149.2 (1.87)		
	rs35316490	A/A	147.6 (1.08)	-6.36 (-10.83 -1.89)	0.005
		A/G-G/G	141.7 (1.96)		
	rs2250312	C/C	148.9 (1.61)	-4.87 (-8.65 -1.09)	0.012
		C/T-T/T	144.4 (1.13)		
	rs4649933	T/T	148.4 (1.23)	-5.5 (-9.22 -1.78)	0.004
		T/C-C/C	143 (1.44)		
	rs2421732	C/C	144.9 (1.06)	4.76 (0.6 - 8.93)	0.025
		C/T-T/T	149.5 (1.94)		
	rs2284362	C/C	148.8 (1.38)	-5.27 (-8.95 -1.61)	0.005
		C/T-T/T	143.7 (1.25)		
	rs2284363	T/T	148.1 (1.11)	-7.41 (-11.52 -3.29)	0.0005
		T/C-C/C	141 (1.68)		
		Diastolic blood pressure (DBP)			
PTGER3	rs7533733	A/A	82.75 (0.82)	-2.16 (-4.17 -0.14)	0.036
		A/G-G/G	80.31 (0.64)		
	rs4649932	A/A	82.4 (0.71)	-2.69 (-4.68 -0.71)	0.008

		A/G-G/G	79.83 (0.74)		
	rs35702222	A/A	82.19 (0.65)	-2.29 (-4.28 -0.31)	0.024
		A/G-G/G	79.88 (0.78)		
	rs115764005	G/G	80.86 (0.53)	4.39 (0.55 - 8.24)	0.025
		G/A-A/A	85.68 (1.93)		
	rs4649933	T/T	82.42 (0.66)	-3.01 (-4.99 -1.02)	0.003
		T/C-C/C	79.44 (0.78)		
	rs2284362	C/C	82.14 (0.69)	-2.06 (-4.02 -0.11)	0.039
		C/T-T/T	80.37 (0.73)		
	rs2284363	T/T	82.04 (0.58)	-3.13 (-5.32 -0.93)	0.005
		T/C-C/C	79.14 (0.98)		
	rs9425054	A/A	81.75 (0.58)	-2.29 (-4.52 -0.07)	0.044
		A/G-G/G	79.54 (1.03)		
	rs77691739	A/A	81.73 (0.56)	-2.92 (-5.52 -0.32)	0.028
		A/G-G/G	78.51 (1.21)		
PTGER4	rs16870224	G/G	81.82 (0.61)	-2.3 (-4.57 -0.03)	0.048
		G/A-A/A	79.5 (0.96)		
			Pulse pressure		
PTGER3	rs6424406	T/T	65.96 (0.86)	-4.68 (-8.70 -0.67)	0.022
		T/C-C/C	60.90 (2.02)		
	rs1536261	C/C	67.46 (1.21)	-3.84 (-6.97 -0.71)	0.017
		C/A-A/A	63.58 (1.06)		
	rs11209708	A/A	65.80 (0.89)	-3.73 (-7.41 -0.06)	0.047
		A/G-G/G	62.42 (1.83)		
	rs1409165	A/A	66.20 (0.96)	-3.85 (-7.16 -0.54)	0.023
		A/G-G/G	62.44 (1.47)		
	rs35316490	A/A	65.85 (0.92)	-4.36 (-8.04 -0.68)	0.021
		A/G-G/G	61.99 (1.62)		
	rs2250312	C/C	66.86 (1.31)	-3.36 (-6.46 -0.26)	0.034
		C/T-T/T	63.67 (0.98)		
	rs2284362	C/C	66.63 (1.13)	-3.21 (-6.23 -0.19)	0.038
		C/T-T/T	63.31 (1.09)		
	rs2284363	T/T	66.04 (0.93)	-4.28 (-7.67 -0.88)	0.014
		T/C-C/C	61.85 (1.47)		

SE, standard error; Mean diff., mean difference with 95% confidence interval

Supplementary Table S4. Cox regression analyses modelling the risk of cardiovascular events in nephrosclerosis patients according to relevant genotypes.

	**B**	**SE**	**Wald**	**HR**	**CI**		**p-value**
rs2241360 AA/AG	-1.051	0.48	4.792	0.35	0.137	0.896	0.029
Sex	1.114	0.441	6.394	3.047	1.285	7.224	0.011
Age	0.052	0.023	5.205	1.053	1.007	1.101	0.023
diabetes	0.619	0.317	3.825	1.857	0.999	3.454	0.05
Hypertension	-0.293	0.731	0.161	0.746	0.178	3.125	0.689
rs7533733 GG	0.871	0.343	6.461	2.39	1.221	4.678	0.011
Sex	0.992	0.413	5.773	2.695	1.2	6.052	0.016
Age	0.043	0.021	4.143	1.044	1.002	1.089	0.042
Diabetes	0.736	0.315	5.446	2.087	1.125	3.871	0.02
Hypertension	-0.077	0.725	0.011	0.926	0.224	3.836	0.916

B, regression coefficient; SE, standard error; HR, hazard ratio; CI, 95% confidence interval.
