# Supplementary figures and images for: Genetic Variants in PGE2 Receptors Modulate the Risk of Nephrosclerosis and Clinical Outcomes in These Patients
Source: J Pers Med. 2021 Aug 6;11(8):772. doi: 10.3390/jpm11080772 (PMC8400263; doi:10.3390/jpm11080772)

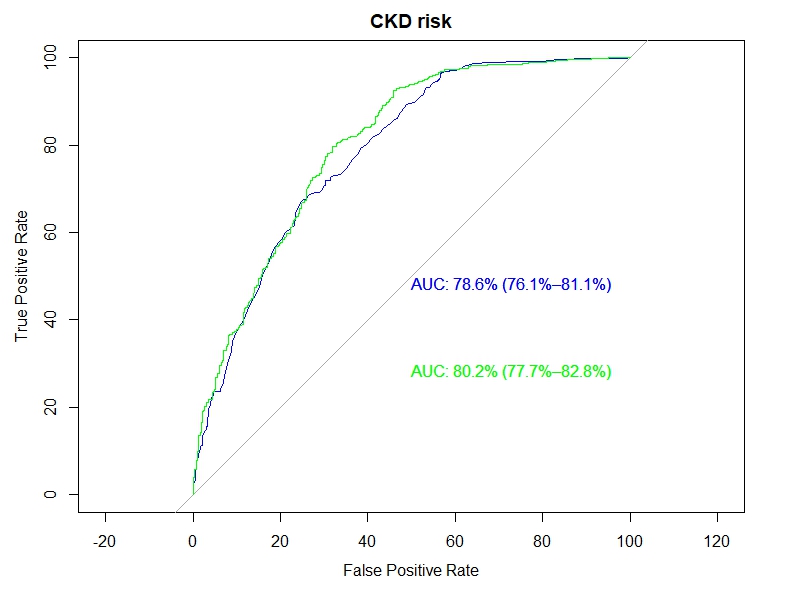

Supplement: Supplementary file 1 [file jpm-11-00772-s001.zip › Suppl Fig S1.jpeg]
